# Chromophobe renal cell cancer - review of the literature and potential methods of treating metastatic disease

**DOI:** 10.1186/1756-9966-28-134

**Published:** 2009-10-07

**Authors:** Rafał Stec, Bartłomiej Grala, Michał Mączewski, Lubomir Bodnar, Cezary Szczylik

**Affiliations:** 1 Department of Oncology, Military Institute of Health Services, Warsaw, Poland; 2 Department of Pathology, Military Institute of Health Services, Warsaw, Poland; 3 Department of Clinical Physiology, Medical Center of Postgraduate Education, Warsaw, Poland

## Abstract

Chromophobe renal cell carcinoma (ChRCC) is a subtype of renal cell carcinoma (RCC). ChRCC is diagnosed mainly in 6th decade of life. An incidence of ChRCC is similar in both men and woman. Eighty six percent of ChRCCs cases are diagnosed in stage 1 or 2. Prognosis of ChRCC is better than in other types of RCC. Five- and 10-year disease free survival (DFS) for ChRCC was 83.9% and 77.9%, respectively. Expression of immunohistological markers: cytokeratins (CK), vimentin, epithelial membrane antigen (EMA), CD10 could be potentially helpful in diagnosis of different subtypes of RCC. From all conventional RCC, CD 117 was detected (overexpression) in membrane of cells ChRCC.

Overexpression of CD117 on cellular membranes of ChRCC could be a potential target for kinase inhibitors like: imatinib, dasatinib, nilotinib. The potential targets for other kinase inhibitors (sunitinib and sorafenib) in ChRCC seem to be VEGFR and PDGFR. On the basis for formulating research hypotheses which should be verified by prospective studies.

## Epidemiology

Renal cell carcinoma (RCC) is rather a rare neoplasm (in Poland about 3% of all tumors). According to the most recent National Cancer Register in Poland, 2150 men and 1501 women were diagnosed with renal cancer in 2004 [1].

Approximately 200,000 new cases of RCC are diagnosed annually worldwide, while the number of deaths caused by RCC approaches 100,000. Cure can be obtained in 70-90% of patients in the TNM stage I, in 55-70% of patients in stage II, in 20-30% of patients in stage III, and in less than 10% in stage IV [2].

The 2004 World Health Organization (WHO) classification of RCC recognized several subtypes of RCC. Most common subtypes are: clear cell RCC (70%), papillary RCC (10-15%), chromophobe RCC (4-6%), collecting duct carcinoma (about 1%) and unclassified RCC (4-5%) [3,4].

Chromophobe RCC (ChRCC) is diagnosed mainly in 6th decade of life. An incidence of ChRCC is similar in both men and woman [5]. 86% of ChRCCs are diagnosed in stage 1 or 2 [3]. Renal vein invasion is seen in about 5% of cases [5].

Incidence of metastatic disease in chromophobe renal cell carcinoma is 6-7% [6,7].

In summary of 28 cases based on 7 reports, most common metastatic sites were liver (39%) and lung (36%) [6].

## Pathology

Chromophobe RCC was first described in patients by Thoenes in 1985 [8].

Macroscopically, ChRCC is a solitary, circumscribed, and not capsulated mass with a homogeneous light brown cut surface. The median tumor size of ChRCC is 6.0 cm, and it is larger than that of other subtypes [7].

Microscopically, it contains of large, polygonal cells with prominent cell membrane [5]. Cytoplasm is pale and resistant to staining with hematoxylin and eosin. ChRCC cells have irregular nuclei with perinuclear clear halo. The tumor blood vessels have thick walls and are eccentrically hyalinized [3].

Chromophobe RCC is a heterogeneous group including classic type, eosinophilic type and mixed type. Eosinophilic variant (containing greater than 80% eosinophilic cells) has areas similar to renal oncocytomas (nested, alveolar or sheetlike architecture with eosinophilic granularity, perinuclear clearing, peripheral accentuation of cytoplasm) and it is often bilateral (11%) and multifocal (22%). Classic type of chromophobe RCC (containing greater than 80% pale cells) is associated with necrosis or sarcomatoid change. It has alveolar or sheetlike architecture and cytoplasm with flocculent "soap-bubble" appearance. Chromophobe RCCs with mixed histology have variable architecture (containing admixture of pale and eosinophilic cells) [6].

Microscopic tumor necrosis and sarcomatoid change are known to be aggressive with a high potential for distant metastases [6].

**Figure 1:**
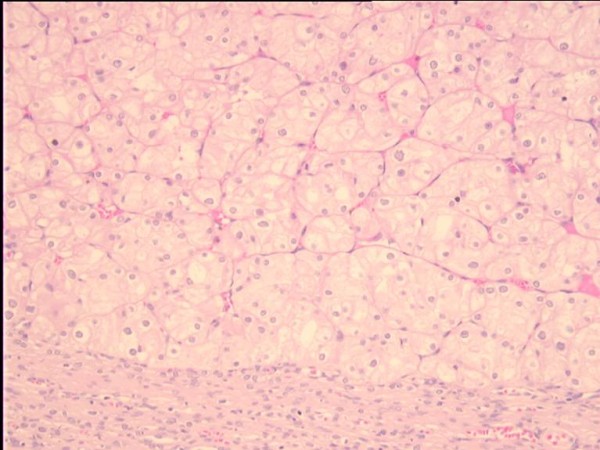
Chromophobe renal cell carcinoma, HE, 200×.

**Figure 2:**
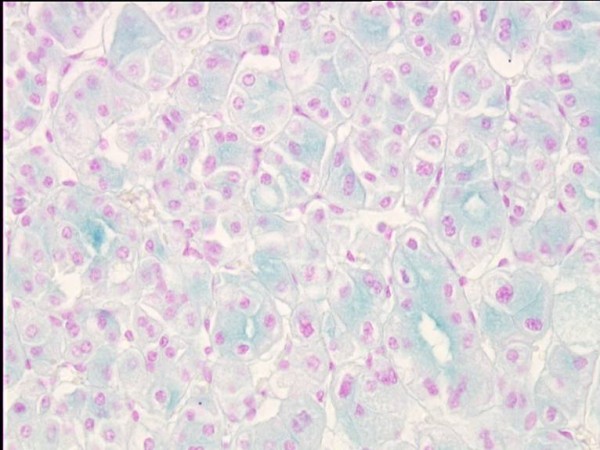
Positive reaction showing the presence of colloidal iron in cytoplasm of ChRCC, 400×.

One of the diagnostic criteria of ChRCC is Hale colloidal iron [5], another are intracytoplasmatic microvesicles between 250-400 nm in diameter [9] (Figure 1- Chromophobe renal cell carcinoma, HE, 200×; Figure 2 - Positive reaction showing the presence of colloidal iron in cytoplasm of ChRCC, 400×).

They can be demonstrated by electron microscopy, which is not used routinely in diagnosis of chromophobe RCC.

The main diagnostic criteria of chromophobe RCC is morphology coupled with characteristic immunophenotype (diffuse CK7, and KIT positivity).

Variable expression patterns of cytokeratins (CK), vimentin, epithelial membrane antigen (EMA), CD10 and parvalbumin can be potentially helpful in diagnosis of different subtypes of RCC. Vimentin was reported positive in 0-21% of ChRCC, CD10 in 0-33% of ChRCC, CK7 in 60-100% of ChRCC, CK8 in 50% of ChRCC, CK18 in 100% of ChRCC, CK19 in 33% of ChRCC, CK20 in 12.5% of ChRCC, EMA 75-100% of ChRCC and parvalbumin 100% of ChRCC. Sometimes ChRCC can be mistaken for renal oncocytoma [10,11] (Table 1).

**Table 1:** Expression of immunohistological markers of ChRCC

**Immunohistological markers of ChRCC**	**CK** **7**	**CK** **8**	**CK** **18**	**CK** **19**	**CK** **20**	**Vimentin**	**EMA**	**CD10**	**Parvalbumin**
**Positive reactivity (%)**	60-100	50	100	33	12.5	0-21	75-100	0-33	100

## Clinical and Histomorphological Features

Prognosis in ChRCC is better than in other types of RCC. Five- and 10-year DFS for chromophobe RCC was 83.9% and 77.9%, respectively [12]. The median time from nephrectomy to metastasis detection, and from metastasis detection to death were twice as long for ChRCC than for other subtypes of RCC (papillary, clear cell RCC) [7].

In univariate analysis: sarcomatoid change (*p < 0.001*), microscopic necrosis (*p = 0.019*), tumor size (*p = 0.025*), pT stage (3.4 vs. 1.2; *p = < 0.001*), broad alveolar growth (*p = 0.012*), vascular invasion (*p = 0.020*), and Fuhrman nuclear grade (grade 4 vs.3 vs 2; *p < 0.001*) were associated with aggressive ChRCC behavior. Independent predictors (Multivariable Cox Regression) of aggressive ChRCC included: pT stage (pT 3.4 vs. pT 1.2; *p = 0.025*, relative hazard 3.4), sarcomatoid change (*p = 0.013*, relative hazard 4.7) and microscopic necrosis (*p = 0.020*, relative hazard 3.5) [6]. Other factors like: age, sex, histologic subtyping by clear, eosinophilic or mixed cell types, tubulocystic pattern, degenerate or symplastic atypia were not predictors of chromophobe RCC behavior.

The patients with aggressive phenotype of chromophobe RCC may be candidates for adjuvant therapies as they become available [6].

ChRCCs are hyperechogenic in ultrasound examination, CT imaging or MRI demonstrate homogeneous enhancement. A spoke-wheel pattern of contrast enhancement is characteristic for ChRCC and for onkocytoma [13]. Most of ChRCCs are sporadic, but sometimes they are associated with BHD (Birt-Hogg-Dubè) syndrome [14].

## Genetic Syndrome associated with chromophobe RCC

BHD syndrome is an autosomal dominant disorder that includes: benign skin tumor (skin tags, fibrofolliculomas), renal epithelial neoplasms (ChRCC, oncocytoma) and spontaneous pneumothorax. Renal tumors are often multifocal and bilateral. BHD gene encodes potential tumor suppressor protein - folliculin on 17p11 [15].

ChRCCs is characterized by length polymorphism such as loss of chromosomal material involving chromosomes: 1, 2, 3p, 6, 10, 13, 17p, 17q and 21 [16,17]. It may be helpful in distinguishing between clear, papillary and chromophobe subtypes of RCC.

## Expression of CD117 (KIT)

KIT (CD117) is a type III receptor tyrosine kinase that plays a role in intracellular signal transduction in several cell types. It regulates apoptosis, cell differentiation, proliferation, chemotaxis, and adhesion. Pathologic activation of KIT through gain-of-function mutations leads to neoplasia of KIT-dependent and KIT-positive cell types in different systems: Cajal cells - gastrointestinal stromal tumors (GISTs), myeloid cells - myeloid leukemia. In addition, many tumors have positive KIT immunoreactivity: small cells carcinomas, adenoid cystic carcinoma, chromophobe, thymic and sometimes ovarian and breast carcinomas [18].

In normal tissue of kidney KIT showed weak immunoreactivity only in the cytoplasm of distal tubules [19]. From all RCCs, *KIT *gene product was detected (overexpression) in membrane of cells ChRCC (88-100%) [19,20]. This is in agreement with histogenetic origin of chromophobe RCC from distal tubules.

KIT expression in classic variant is more often than eosinophilic variant (82% vs. 67%) [21].

Thus, immunohistochemical detection of KIT expression appears to be useful in diagnosis and treatment of ChRCC.

Yamazaki et al. reported upregulation of *c-kit *gene expression in ChRCCs. The mechanism for the overexpression of KIT in ChRCC is unknown. They suggested that the KIT signal pathway in ChRCCs could be activated in an autocrine way [19]. In summary 70 cases, based on 4 reports investigators were unable to detect activating mutations within exon 17 of the *c-kit *gene [19-22]. Absence of *c-kit *mutation could be argue for potential effectiveness of imatinib therapy in patients with metastatic ChRCCs.

## Potential targeted therapy for advanced ChRCC

Now we have three potentially active and targeted agents against CD 117: imatinib, dasatinib and nilotinib.

### Imatinib

as KIT tyrosine kinase inhibitor (TKI) is an accepted treatment of chronic eosinophilic leukemia, hypereosinophilic syndrome, chronic myeloid leukemia, myelodysplastic/myeloproliferate syndrome, acute lymphoblastic leukemia, dermatofibrosarcoma protuberans, gastrointestinal stromal tumors [18]. The targets for imatinib include: BCR/ABL, CD 117, PDGFRA (platelet-derived growth factor receptor) [23] and also DDR1 (discoidin domain receptor 1), NQO2 (quinone reductase QR2) [24,25].

### Dasatinib

is a second-line multikinase (besides BCR/ABL kinase) inhibitor. Dasatinib is used in patients with chronic myeloid leukemia or acute lymphoblastic leukemia with resistance or intolerance of imatinib. *In vitro*, it has approximately 325-fold greater potency than imatinib in inhibition of BCR/ABL kinase [26]. In phase II trial, dasatinib increased response rates by > 2-fold versus high-dose of imatinib. The targets for dasatinib include: BCR/ABL, CD 117, PDGFRA, DDR1, DDR2, Src family kinases and ephrin receptor kinases [24,27].

### Nilotinib

is the result of modifications to the imatinib molecule [28,29]. Nilotinib like imatinib, inhibits BCR/ABL, CD 117, PDGFRA, NQO2, DDR1 [24,25,29]. Nilotinib also inhibits CSF-1R (colony-stimulating factor-1 receptor) [30] and EphB4 (ephrin receptor) [31]. Nilotinib was 43-60 times more potent than imatinib in cell lines (KBM5, KBM7) [32].

A phase II clinical trial confirmed activity of nilotinib in imatinib-resistant or imatinib-intolerant chronic myeloid leukemia [33] (Table 2).

**Table 2:** Targets for Imatinib, Dasatinib and Nilotinib

**Target spectrum**	**Imatinib**	**Dasatinib**	**Nilotinib**
BCR-ABL	+	+	+

PDGFR	+	+	+

c-KIT	+	+	+

Src family kinases	-	+	-

Ephrin receptor kinases	-	+	only EphB4

NQO2	+	-	+

DDR1	+	+	+

CSF-1R	-	-	+

We realize that this treatment hypothesis is controversial. Up to now, we have not found cases of successful treatment in the literature. But we think, that prospective trials with these agents in ChRCC should clarify their use in the future.

Other interesting therapies for advanced ChRCC may include therapies used in advanced clear cell renal carcinoma (CCRCC). Both, **sorafenib **and **sunitinib **showed clinical activity in randomized clinical trials in treatment metastatic CCRCC [34,35]. These are tyrosine kinases inhibitors including vascular endothelial growth factor receptor (VEGFR) and platelet-derived growth factor receptor (PDGFR) [36,37].

VEGF and PDGF are markers of angiogenesis which plays an essential role in tumor growth and metastatization. Overexpression VEGF and PDGF in RCCs is associated with defective von Hippel-Lindau (VHL) protein. It can induce the expression of the genes involving in angiogenesis through the hypoxia-inducible factor 1α (HIF-1α) pathway. *VHL *is inactivated in up to 80% of sporadic cases of clear-cell carcinoma [38].

ChRCC can be associated with high serum levels of VEGF, making VEGF-targeted therapy an attractive therapeutic option [39].

In biochemical and cellular tests both agents inhibit CD 117. They seem to be next potential targeted therapy for advanced ChRCC [37].

Choueiri et al. confirmed, that sunitinib and sorafenib are active agents in metastatic ChRCC: 75% of patients had stable disease (SD) more than 3 months and 25% had partial response (PR) [37] Table 3.

**Table 3:** Activity Sorafenib and Sunitynib in ChRCC

**Agent**	**No. of patients**	**Median PFS (months)**	**Partial Response** **No. of patients**	**Stable Disease** **No. of patients**
Sunitinib	7	8.9	1	6

Sorafenib	5	27.5	2	3

## Conclusion

Currently, we do not have any effective treatment for the metastatic disease apart from surgical procedures. Overexpression of CD117 on cellular membranes of ChRCC could be a potential target for kinase inhibitors like: imatinib, dasatinib, nilotinib. The potential targets for other kinase inhibitors (sunitinib and sorafenib) in ChRCC seem to be VEGFR and PDGFR. In conclusion, these observations are the basis for formulating research hypotheses which should be verified in prospective studies.

## Competing interests

The authors declare that they have no competing interests.

## Authors' contributions

RS, LB, MM participated in the sequence alignment and drafted the manuscript. BG was responsible for pathomorphology. RS, CS was responsible for coordination. All authors read and approved the final manuscript.
